# Measuring High Dynamic Range Spectral Reflectance of Artworks through an Image Capture Matrix Hyperspectral Camera

**DOI:** 10.3390/s22134664

**Published:** 2022-06-21

**Authors:** Ángela Gómez Manzanares, Daniel Vázquez Moliní, Antonio Alvarez Fernandez-Balbuena, Santiago Mayorga Pinilla, Juan Carlos Martínez Antón

**Affiliations:** Department of Optics, University Complutense of Madrid, 28040 Madrid, Spain; anggomez@ucm.es (Á.G.M.); dvazquez@ucm.es (D.V.M.); smayorga@ucm.es (S.M.P.); jcmartin@ucm.es (J.C.M.A.)

**Keywords:** hyperspectral image, spectral reflectance, high dynamic range, artwork, filter matrix

## Abstract

Commercial hyperspectral imaging systems typically use CCD or CMOS sensors. These types of sensors have a limited dynamic range and non-linear response. This means that when evaluating an artwork under uncontrolled lighting conditions and with light and dark areas in the same scene, hyperspectral images with underexposed or saturated areas would be obtained at low or high exposure times, respectively. To overcome this problem, this article presents a system for capturing hyperspectral images consisting of a matrix of twelve spectral filters placed in twelve cameras, which, after processing these images, makes it possible to obtain the high dynamic range image to measure the spectral reflectance of the work of art being evaluated. We show the developed system and describe all its components, calibration processes, and the algorithm implemented to obtain the high dynamic range spectral reflectance measurement. In order to validate the system, high dynamic range spectral reflectance measurements from Labsphere’s Spectralon Reflectance Standards were performed and compared with the same reflectance measurements but using low dynamic range images. High dynamic range hyperspectral imaging improves the colorimetric accuracy and decreases the uncertainty of the spectral reflectance measurement based on low dynamic range imaging.

## 1. Introduction

The light reflected from an object is the combination of the reflectance spectral properties of the object itself and the incident illumination [1]. When light strikes the visual system, it is perceived by the human eye, creating the perception of color [2]. Unlike other factors, such as illumination and the observer, reflectance is an inherent property of the object [3].

The analysis of objects through spectral reflectance measurements is widely applied and used in agriculture [4], medicine [5], criminology [6], and the restoration and conservation of artworks [7,8] because it is possible to show aging in objects due to spectral reflectance variations. The International Commission on Illumination (CIE) recommends different geometrical conditions for the sample, the illumination, and the measuring instrument in reflectance measurements [9]. Depending on these conditions, different types of reflectance are classified [2,9,10]. Among the proposed methods by the CIE to measure spectral reflectance, an integrating sphere is recommended to ensure that the sample is uniformly illuminated from all angles [10,11]. However, when working with artwork, Fairchild recommends using the bidirectional 0–45º geometry proposed by the CIE, in which the receiver is located at the normal with respect to the sample to be tested and the emitter is located at 45° with respect to the normal. This geometric configuration should be considered with other methods because it is not ideal for illuminating from all angles, and it is desirable to discard the possible brightness of the pigments evaluated so that the specular component of the reflection is not considered. Furthermore, this geometric configuration emphasizes the texture of the material to be evaluated [2,9]. For this purpose, the 0–45° configuration is recommended, in which the receiver is located at the normal with respect to the sample to be tested, and the emitter is located at 45° with respect to the normal [2].

To avoid contact with the artwork and not damage it, image spectrophotometers are used [10,12,13]. Nevertheless, although the energy obtained from the sample is very detailed, the measurement area of the object is approximately 5 mm in diameter [3,10]. Thus, to measure larger areas, the technique for obtaining diffuse spectral reflectance from hyperspectral imaging has been extended in different sectors for the analysis of objects without spatial limitation [4,7,14]. Hyperspectral and multispectral cameras are defined by the spectral resolution being higher in hyperspectral ones [15,16]. In the context of restoration and conservation of cultural heritage, this technique is very interesting because it not only allows contact with the artwork to be avoided but also allows spectral evaluation with more extensive spatial information [7,17]. Mona F Ali et al. used multispectral imaging technology, among other optical technologies, to evaluate cartonnage fragments from the Egyptian Museum in Cairo [18]. As Bratitsi et al. state in their study, multispectral and hyperspectral imaging offers great advantages for the identification of pigments in artworks [19]. This technology is also used, among others, in aerial and remote sensing archaeology for imaging large areas [20].

The technique for obtaining diffuse spectral reflectance from hyperspectral imaging consists of calculating the spectral reflectance of an image with pixel precision through the collection of hyperspectral images in a number of continuous spectral bands [7,21]. To carry out the measurements, a hyperspectral image of the object is compared with a hyperspectral image of a perfectly reflected diffuse material without varying the geometrical and illumination conditions between the two situations [2]. The set of spectral images obtained is called the spectral reflectance image-cube. Two dimensions of the hyperspectral reflectance image-cube correspond to the spatial dimensions of the image (m×n pixels), and the third dimension corresponds to the wavelength [7].

The high costs of commercial hyperspectral systems [22] have led to the development of alternative hyperspectral systems that are not only cheaper but as accurate as the commercial systems. Among them, the use of a wheel with filters at different wavelengths coupled to a monochrome camera stands out. In this way, spectral images are captured sequentially, finally obtaining an image with the spectral range corresponding to the number of filters used [23]. Changying Li and Weilin Wang explore hyperspectral imaging systems using liquid crystal tunable filter (LCTF) technology for vegetable analysis. LCTF technology allows for control of the transmitted light by selecting the transmission wavelength and excluding the rest. Therefore, these systems allow hyperspectral imaging without the need to exchange spectral filters between measurements [24]. Baek et al. designed a hyperspectral imaging system using dispersion on the image obtained through prisms [25]. Other studies optimized the spectral information of LED light sources by obtaining sequences of spectral bands for capturing each spectral image [26]. Geelen et al. developed a hyperspectral imaging system by modifying the Bayer matrix of the sensor used. In their experiment, they use optical filters that are placed monolithically on the pixels of the camera sensor. In this way, they achieve a system with an increase in the spectral resolution of the camera, without modifying the temporal resolution of image capture with respect to RGB cameras [27]. N. Genser et al. proposed an array of cameras, each with a spectral filter, for hyperspectral imaging [28].

Hyperspectral imaging systems typically use charge-coupled device (CCD) or Complementary Metal-Oxide-Semiconductor (CMOS) image sensors, which are limited in their dynamic range and have a non-linear response [29]. In the evaluation of artworks through hyperspectral imaging, this limitation is reflected when the artwork itself has a high dynamic range in its scene. The hyperspectral image obtained from that scene will have areas outside the linear dynamic range of the sensor, showing saturated or underexposed pixels [30]. On the other hand, the uniformity of the illumination on an artwork is not always controlled [31]. Even if the artwork does not have such an energetic variation in the spectral reflectance of its pigments, this variation in the energetic contribution to the scene can generate a limitation in the dynamic range of the hyperspectral image that is obtained. To solve this problem, high dynamic range (HDR) images have been used. This technique consists of combining several images of the same scene at different exposure times [32].

In 2008, J. Brauers et al. combined hyperspectral technology with HDR technology for the first time by using a spectral filter wheel system coupled to an RGB camera [29]. However, hyperspectral image capture systems using a filter wheel capture each hyperspectral image sequentially [29], which increases the capture time compared to non-sequential systems. This combination has also been used by M. A. Martinez et al., who obtained spectral reflectance through HDR hyperspectral imaging using a hyperspectral line scanner [31].

In this study, we developed a hyperspectral imaging system consisting of a matrix of 12 cameras with one spectral filter fitted on each camera, with a resolution of 4 K and a color depth of 12 bits. The post-processing of these hyperspectral images allows HDR hyperspectral images to be obtained, which are then used to obtain the high dynamic range spectral reflectance. The developed system, called Hypermatrixcam, has been designed to evaluate the spectral reflectance parameter of artworks in order to obtain objective and high-quality spectral and colorimetric information on the state of conservation of these works if measured through time.

## 2. Materials and Methods

### 2.1. Experimental Set-Up

**Figure 1:**
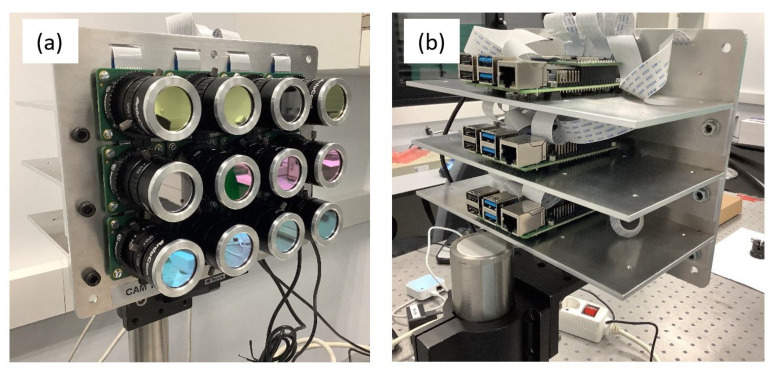
(**a**) Front of the Hypermatrixcam where it is possible to see the spectral filters used attached to the Raspbery pi HQ cameras. (**b**) The back view shows the configuration of the three Raspberry Pi 4 used to control the 12 cameras.

In this study, we developed a system of twelve cameras arranged in a matrix array (3 × 4). Each camera has a spectral band-pass filter to obtain hyperspectral images (Figure 1a). The developed system aims to capture hyperspectral images with as much signal as possible, optimizing the acquisition time so that light exposure on the artworks is minimized during the measurements. The time optimization of the image capture was performed using three Raspberry pi 4. Each Raspberry pi controls four cameras using Arducam’s Multi-camera adapter board v2.2 multiplexers. This reduces the capture time by a factor of 3 (Figure 1b).

**Figure 2:**
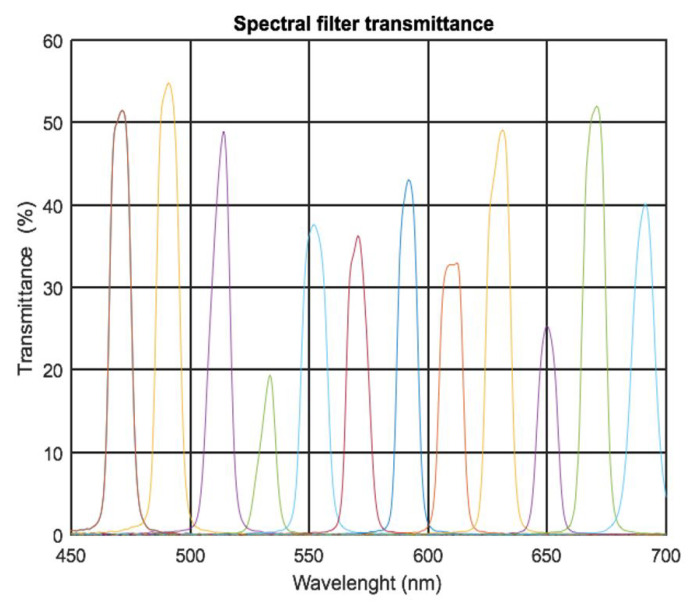
Transmittance curve of the spectral filters ordered left to right of 470, 490, 510, 530, 550, 570, 590, 610, 630, 650, 670, and 690 nm.

The cameras used were Raspberry pi HQ cameras consisting of Sony IMX477 RGB CMOS sensors. They can obtain images with a resolution of up to 4K (4056 × 3040 px.) and a color depth of 12 bits. Although the use of RGB sensors compromises the amount of signal obtained in some wavelengths with respect to monochromatic sensors, RGB sensors have better performance in terms of resolution and color depth and are also affordable both economically and commercially. The cameras are mounted with 12 mm focal length lenses from Arducam. This lens model is specifically designed for this model of Raspberry Pi camera to avoid possible vignetting problems. At the minimum focusing distance (0.2 m), a resolution of 50 pixels/mm of the artwork is obtained. Each camera has a field of view of 30° horizontally and 22.7° vertically, so considering the spatial displacement between cameras, the Hypermatrixcam can cover a field of view of 23.4° × 20.5° (H × V). We designed a mechanical steel structure that allowed the spectral filters to be screwed onto the lenses. This system offers the advantage of being able to easily exchange the selected spectral filters depending on the study to be carried out. The spectral band-pass filters are from Thorlabs [33]. Because the artworks will be viewed by the human eye, the selection of the spectral filters was made according to the sensitivity curve of the human eye (V(λ)). Taking into account the RGB cameras used, the range of the visible spectrum between 470 and 690 nm in steps of 20 nm was selected in order to cover the range of the maximum spectral signal of the cameras with the continuous spectrum. The transmittance curve of the 12 filters (Figure 2) was measured using a fiber-optic spectrophotometer (HR Spectrophotometer from StellarNet Inc., Tampa, FL, USA).

**Figure 3:**
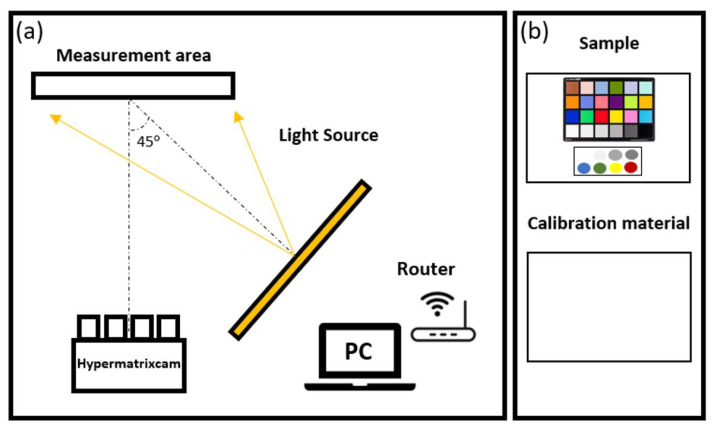
(**a**) Schematic of the measurement procedure to obtain the spectral reflectance using HDR hyperspectral imaging. The light source is placed at 45° with respect to the measurement device (Hypermatrixcam). The samples to be tested (in this case, the Colorchecker (**b**) and the calibration target (**b**) are placed in the measurement area.

Because illumination causes photochemical damage to the artwork [34], the light source chosen for this application is an LED system that produces almost no heat, has no UV or IR light, and is now widely used in museums [35]. In our system, we chose a 610 × 410 mm LED array as the light source. This configuration provides greater uniformity over the sample to be tested, reducing the effects of specular reflection that could alter the measurements. As recommended by Fairchild in [2], in our study, we chose the 0–45° configuration, as shown in Figure 3. In this configuration, the light source was placed with its geometric center at 45° from the center of the measurement area with respect to the normal, where the Hypermatrixcam was located. The Hypermatrixcam is wireless. The measuring distances were not fixed. Only the field of view of the Hypermatrixcam must be taken into account, and the illumination must be at an angle of 45° to the measuring area.

### 2.2. Measurement Procedure

#### 2.2.1. Calibration

The spectral reflectance measurement procedure using hyperspectral imaging was performed according to Equation (1). The spectral reflectance was calculated as $\rho_{S}\left(\lambda \right)$ at each pixel *n*, *m*:

(1) $$\rho_{S}{\left(\lambda \right)}_{\left(n,m\right)}=\rho_{white}{\left(\lambda \right)}_{\left(n,m\right)}\frac{I_{S}{\left(\lambda \right)}_{\left(n,m\right)}-I_{dark}{\left(\lambda \right)}_{\left(n,m\right)}}{I_{white}{\left(\lambda \right)}_{\left(n,m\right)}-I_{dark}{\left(\lambda \right)}_{\left(n,m\right)}},$$

where $I_{S}\left(\lambda \right)$ is the hyperspectral image of the sample, $I_{white}\left(\lambda \right)$ is the hyperspectral image of the reference material (typically white) needed to perform the spectral reflectance calculations, $\rho_{white}$ is the spectral reflectance value of the reference material, and $I_{dark}\left(\lambda \right)$ is the dark image necessary to calibrate the sensor noise and background, which was obtained by capturing the hyperspectral image with the sensors covered.

**Figure 4:**
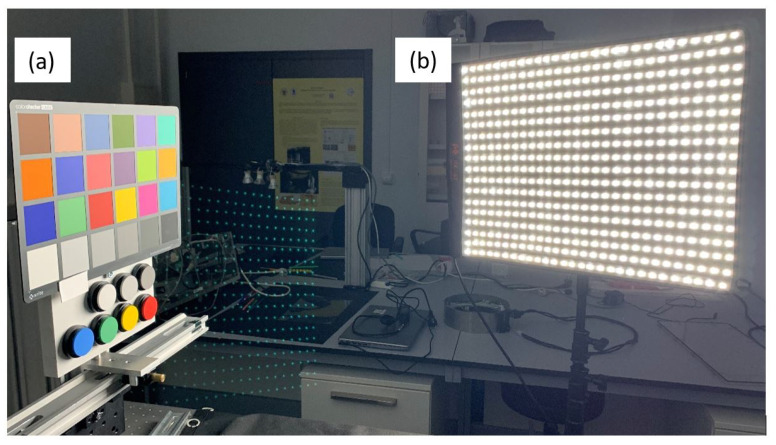
(**a**) The sample measured in this experiment consisting of a Colorchecker (classic model) and seven Labsphere’s Spectralon Reflectance Standards. (**b**) Illumination source used with a magnitude of illuminance of 1000 lux on the sample.

Once the geometry was defined, as shown in Figure 3a, the hyperspectral image of the calibration sample ($I_{white}\left(\lambda \right)$) (Figure 3b) was captured at various exposure times. The material used for this measurement was foamed polyvinyl chloride (PVC) for its Lambertian and achromatic properties. Its spectral reflectance was measured using an integrating sphere colorimeter (CM2600d). Then, without changing the position of the light source or the instrument used, the hyperspectral image of the sample to be tested ($I_{S}\left(\lambda \right)$) was taken (Figure 3b). These measurements were performed with the same exposure times as the $I_{white}\left(\lambda \right)$ measurements. Both the sample and the reference white material were positioned at 1.1 m from the Hypermatrixcam for the measurements. This distance was selected to obtain the highest resolution over the measurement area considering the field of view of the Hypermatrixcam. At each exposure time, the dark current $I_{dark}\left(\lambda \right)$ was also imaged, which was subtracted from each $I_{S}\left(\lambda \right)$ and $I_{white}\left(\lambda \right)$ image. In order to validate the system, seven samples of Labsphere’s Spectralon Reflectance Standards and a Colorchecker card were measured (Figure 4). In order to validate the system under the same geometrical and illumination conditions, all samples were measured under the same geometrical and illumination conditions by a non-contact spectrophotometer (spectrophotometer PR655) despite the manufacturer already providing the spectral reflectance values of the materials.

**Figure 5:**
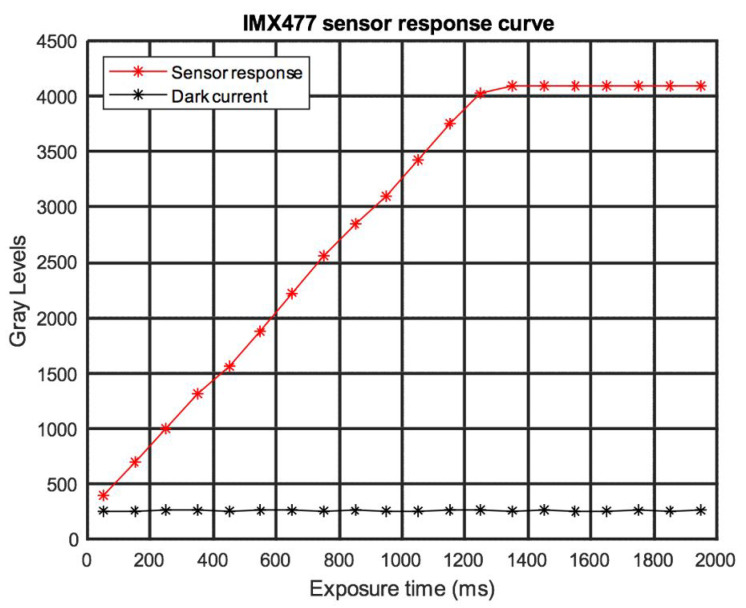
Response curve of the IMX477 sensor.

The Raspberry Pi HQ cameras have CMOS type-sensors and a color depth of 12 bits, so their dynamic range is limited to 4096 grey levels. The response curve of the camera as a function of exposure time is shown in Figure 5.

This measurement was made by taking an area of the image captured by one of the twelve sensors used, and the average of the grey levels in the selected area was calculated for each exposure time between 50 and 1900 ms. The nonlinearity zone of the camera starts at 3750 grey levels, and the dark current of the sensors is below 550 grey levels.

#### 2.2.2. HDR Algorithm

**Figure 6:**
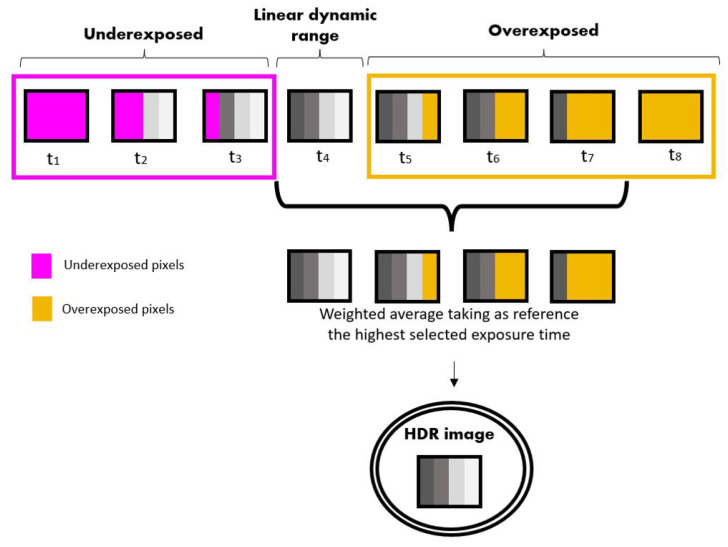
LDR image processing scheme to obtain HDR images.

The exposure times were selected so that in at least one of the images, the pixel value was within the linear zone of the dynamic range of the sensor (Figure 6). The rest of the selected images must be in the overexposed zone, with the areas of lower dynamic range of the image within the linear zone of the sensor. In the development of this system, we took 4 images. Next, the weighted average of the selected images was taken with respect to the image with the longest exposure time. To perform this measurement, the pixels whose grey level values were within the linear zone of the dynamic range of the camera were selected. As shown in Figure 5, in the development of this algorithm, the upper limit is 3500 grey levels and the lower limit is 300 grey levels. The lower limit was taken as 300 grey levels because it is the value of the dark current of the sensors, measured by taking the image with the sensors covered. The result of this process is an HDR image for the RGB channel, which is then summed.

The mathematical procedure is described in Equation (2):

(2) $$HDR\left(n,m\right)=\frac{\left(\frac{t_{1}}{t_{q}}\right)LDR{\left(n,m\right)}_{1}+\left(\frac{t_{2}}{t_{q}}\right)LDR{\left(n,m\right)}_{2}+\ldots +\left(\frac{t_{q}}{t_{q}}\right)LDR{\left(n,m\right)}_{q}}{q},$$

where *LDR (n,m)* corresponds to the low dynamic range image with spatial dimensions of *n* × *m* pixels at a given exposure time *(t)*, and *q* corresponds to the number of images selected to create the HDR image. Finally, *HDR (n,m)* corresponds to the HDR image with spatial dimensions of n x m pixels. Once the HDR image was obtained for the sample and for the calibration target measurements, the HDR spectral reflectance was calculated from Equation (1), considering that $I_{dark}\left(\lambda \right)$ has been previously subtracted from each measurement.

## 3. Results

**Figure 7:**
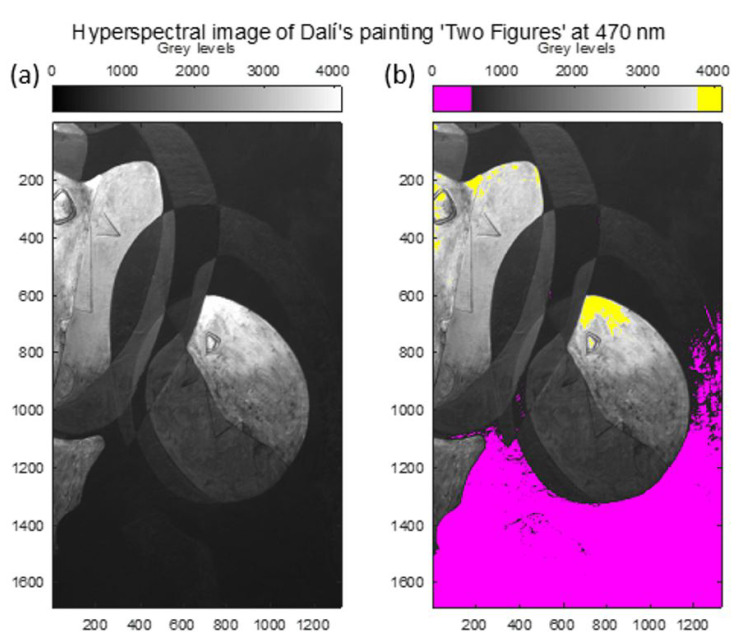
(**a**) Low dynamic range hyperspectral image of an enlarged area of Salvador Dalí’s painting “Two Figures”. (**b**) Hyperspectral image pixels saturated (yellow) and underexposed (pink).

The artwork “Two Figures (1926)” by Salvador Dalí has a high dynamic range in its scene. Figure 7a shows the low dynamic range hyperspectral image of the enlarged area of the painting. In Figure 7b, both saturated (yellow) and underexposed (pink) areas can be seen in the same image.

**Figure 8:**
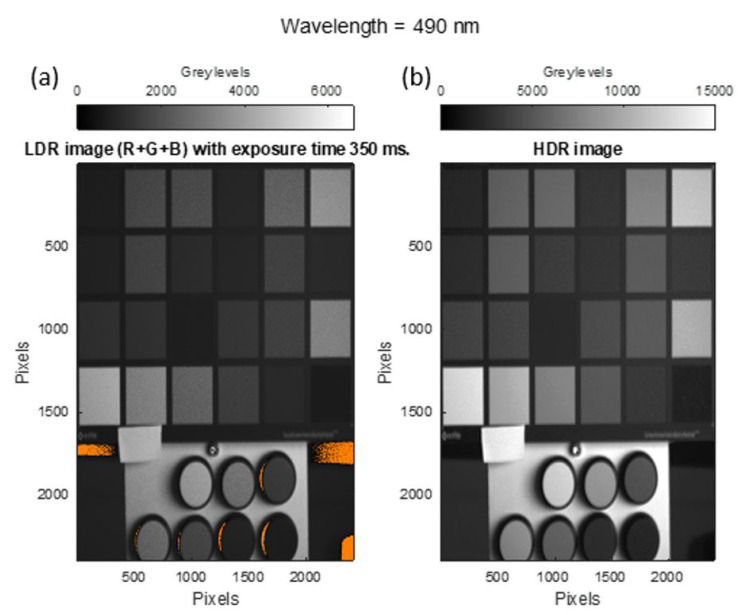
(**a**) LDR image and (**b**) HDR image at 490 nm. Orange shows pixels whose signal has one of the RGB channels outside the linear zone of the sensor.

Figure 8 shows the HDR image (a) and the LDR image (b) at 490 nm. For the LDR image, images with an exposure time of 350 ms were used. In the LDR image (Figure 8a), the areas whose signal (sum of the three RGB channels) is lower than the linear area of the dynamic range of the sensor can be seen in orange.

For the development of the HDR hyperspectral reflectance cube, several exposure times were taken for each spectral camera. In order to assess the limits of the camera’s dynamic range, measurements were initially taken with exposure times between 50 and 30,100 ms. Measurements were taken at these exposure times in order to obtain a signal throughout the selected range of 470 to 690 nm. It was observed that for the 1000 ms exposure time, sensors with spectral filters between 470 and 590 nm showed the image to be fully saturated. However, sensors from 590 to 690 nm needed a higher exposure to reach saturation in their images. This is due to the influence of the transmittance of the spectral filters in combination with the difference in the spectral sensitivity of the RGB sensors used. The selected exposure times for each measurement varied from 150 to 30,100 ms in steps of 300 or 1000 ms, depending on the filter used. Once the hyperspectral images of the sample and the white material used for calibration (Figure 3b) were obtained, the spectral reflectance cube was obtained. Figure 8 shows the spectral reflectance of the HDR spectral reflectance cube at 490 nm.

**Figure 9:**
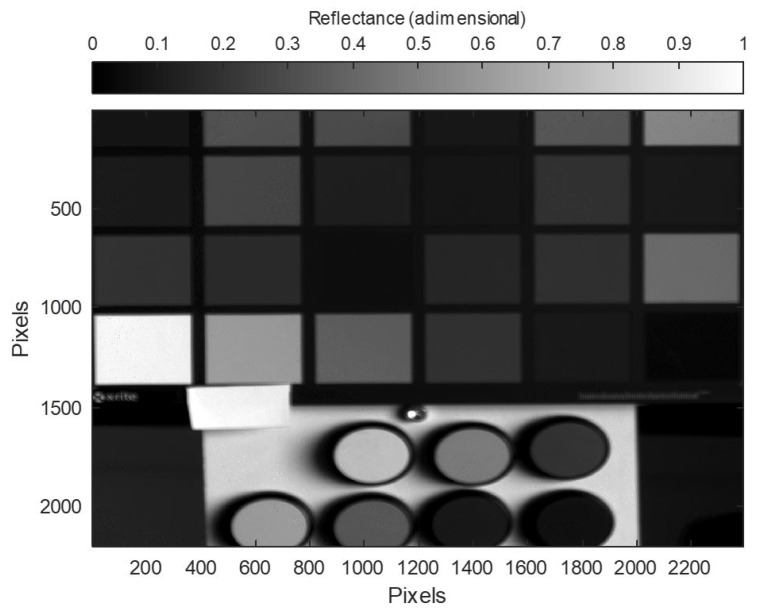
HDR reflectance at 490 nm.

To validate the system, the HDR spectral reflectance measurement obtained was compared with the low dynamic range spectral reflectance. For this purpose, both spectral reflectance measurements were compared with those obtained using a spectrophotometer (model PR-655). The spectrophotometer used takes a measuring area of 5 mm in diameter and integrates the radiance values obtained in the sample. Subsequently, as with the hyperspectral images, Equation (1) was used to obtain the spectral reflectance measurement. In addition, the HDR spectral reflectance measurements were also compared with the spectral reflectance provided by the manufacturer Labsphere of the reflectance standards used. As an example (Figure 9) shows the HDR reflectance at 490 nm where all pixel reflectance is processed to be inside linear behaviour.

**Figure 10:**
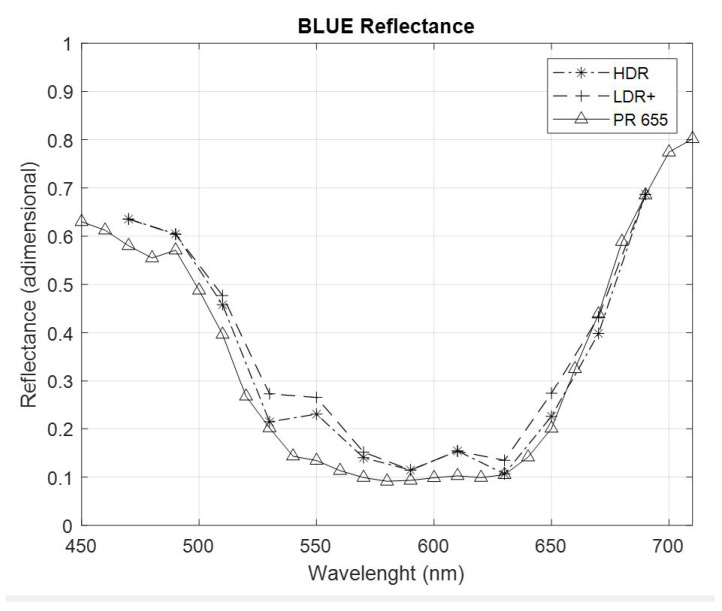
Spectral reflectance of the Labsphere’s blue reflectance standard through hyperspectral imaging of HDR, LDR+, and the PR 655 spectrophotometer.

Figure 10 shows the spectral reflectance of the Labsphere blue reflectance standard. It shows the LDR spectral reflectance, the HDR spectral reflectance, and the spectral reflectance obtained from the PR 655 spectrophotometer. The LDR+ curve shown corresponds to the LDR values with optimized exposure times for each sensor, selecting the maximum exposure time that obtains the maximum signal values within the linear dynamic range of the sensor. Due to the difference in the spectral sensitivity of the RGB sensors, each of these sensors will have its maximum linear dynamic range limit at a specific exposure time. Therefore, to evaluate the spectral reflectance through LDR imaging, it is necessary to select the exposure times individually for each sensor, with the goal of obtaining the highest possible signal at the pixels with maximum value as long as these grey level values remain within the linearity zone of the sensor’s dynamic range (Figure 5).

In order to evaluate the colorimetric accuracy and measurement error of the system, Table 1 evaluates the color difference ($\Delta E_{ab}^{*}$) and root mean square (RMS) value of the LDR spectral reflectance and HDR spectral reflectance for the red, green, and blue standards. Both parameters were evaluated with respect to the PR655 spectrophotometer and the reflectance curves of the Labsphere’s reflectance standards used.

## 4. Discussion

This article presents a high dynamic range spectral reflectance measurement method that combines HDR technology and the development of a system capable of obtaining hyperspectral images with high spatial resolution developed from devices that are economically and commercially accessible. In this way, it facilitates its use without the need for a great deal of prior knowledge of electronics.

Unlike hyperspectral cameras based on a filter wheel, the system described in this study allows hyperspectral imaging without the need to move spectral filters at each measurement [29]. Although this disadvantage is overcome by the use of LCTF technology, both LCTF technology and filter wheels obtain hyperspectral images sequentially, affecting the time of image acquisition in both cases [24]. The system described by Geelen et al. allows the capture of hyperspectral images without changing the temporal resolution with respect to RGB imaging. However, unlike Hypermatrixcam, when using the method described by Geelen et al., the spatial resolution of the sensor used is reduced as the spectral resolution of the system increases [27]. A hyperspectral imaging matrix system was proposed by N. Genser et al. [28] that obtained the spectral reflectance using low dynamic range spectral images. Unlike their system, in this study, we used the matrix configuration to obtain HDR spectral reflectance. Martínez et al. obtained the spectral reflectance parameter by means of HDR hyperspectral imaging using a hyperspectral line scanner [31]. This configuration has the disadvantage that in order to evaluate the artwork, it is necessary to move the scanner along its length. In our study, it was possible to obtain the HDR spectral reflectance through hyperspectral imaging, obtaining the spectral reflectance parameter pixel by pixel with an area the size of the captured image. The measurements described in this paper were performed in a laboratory under sufficient illuminance conditions in all areas of the evaluated scene. Thus, using the exposure time that defines the maximum limit of the line response zone of the sensor, the signal in the low dynamic range zone was sufficient to obtain the reflectance results. However, as in the analysis of Goya’s painting “Portrait of Juan Martin de Goicoechea” [30], when evaluating the spectral reflectance of artworks, the illuminance over the scene is not sufficient in low dynamic range areas, so in this case, using the HDR hyperspectral images to evaluate the spectral reflectance is an indispensable requirement.

## 5. Conclusions

In this study, a 4 K resolution hyperspectral image capture matrix system capable of obtaining high dynamic range spectral reflectance was developed. The system was developed from commercially available devices, facilitating its manufacture and cost. The matrix configuration of the cameras used allows images to be captured without needing to exchange spectral filters between measurements because a spectral filter at a pre-selected wavelength is placed on each sensor, which implies a reduction in the radiation on the artwork. The flexibility of the Hypermatrixcam means that by changing the band-pass filters, depending on the characteristics of the CCDs used, it is possible to work in the spectral range from the UV to the near IR, making it a very appropriate device for hyperspectral measurements. According to the results obtained, both the color difference parameter and the root mean square provide improvements in HDR over LDR+, even under controlled lighting conditions. Referring to the spectral reflectance measurements obtained with the PR-655 spectrophotometer, ΔE and RMS show improvements of 22.5% and 21.4%, respectively. If the Spectralon standard reflectance curve is taken as a reference, the improvements in ΔE and RMS are 24.53% and 15.38%, respectively. For the evaluation of artworks where lighting conditions are not always controlled, the described method allows for the evaluation of areas of the artwork that could not have been evaluated without HDR.

## Figures and Tables

**Table 1:** $\Delta E_{ab}^{*}$ and RMS of the LDR spectral reflectance and HDR spectral reflectance for the red, green, and blue patterns with respect to the measurements obtained through the PR655 spectrophotometer and the calibrated reflectance curves provided by the manufacturer.

	Comparison with Spectral Reflectance Obtained with PR 655				Comparison with Spectral Reflectance Provided by the Manufacturer			
	$\Delta E_{\mathrm{LDR}+}$	$\Delta E_{\mathrm{HDR}}$	${\mathrm{RMS}}_{\mathrm{LDR}+}$	${\mathrm{RMS}}_{\mathrm{HDR}}$	$\Delta E_{\mathrm{LDR}+}$	$\Delta E_{\mathrm{HDR}}$	${\mathrm{RMS}}_{\mathrm{LDR}+}$	${\mathrm{RMS}}_{\mathrm{HDR}}$
Red	17.09	14.04	0.014	0.013	17.37	14.25	0.015	0.014
Green	7.03	6.69	0.012	0.006	4.17	3.42	0.013	0.006
Blue	10.68	6.24	0.018	0.013	4.27	1.79	0.011	0.012
Mean	11.6	8.99	0.014	0.011	8.60	6.49	0.013	0.011

## Data Availability

Data available on request to the corresponding author due to restrictions of museum data related to Dali’s painting.
